# Genome-Wide Association Study of Six Forage Traits in Ramie (*Boehmeria nivea* L. Gaud)

**DOI:** 10.3390/plants11111443

**Published:** 2022-05-28

**Authors:** Xuehua Bai, Xin Wang, Yanzhou Wang, Yiping Wei, Yafen Fu, Jing Rao, Yonghong Ma, Zheng Zeng, Fu Li, Mansheng Wang, Siyuan Zhu

**Affiliations:** Institute of Bast Fiber Crops, Chinese Academy of Agricultural Sciences, Changsha 410205, China; 18235707017@163.com (X.B.); 2019011129@stu.gxnu.edu.cn (X.W.); wangyanzhou@caas.cn (Y.W.); weiyiping@hnyesfgdzkxx1.wecom.work (Y.W.); fuyafen_1995@hotmail.com (Y.F.); jingrao@oebiotech.com (J.R.); yonghongma@annoroad.com (Y.M.); 82101121030@caas.cn (Z.Z.); lifu@webmail.hzau.edu.cn (F.L.); wangmansheng@caas.cn (M.W.)

**Keywords:** ramie, forage trait, genome-wide association study, single nucleotide polymorphisms

## Abstract

Genome-wide association study (GWAS) of six forage traits using whole-genome sequencing data generated from 301 ramie accessions found that traits were continuously distributed; the maximum variant coefficient was fresh weight per clump (FWPC) (2019) and individual plant height (IPH) (2019) minimum. Correlation analysis demonstrated that 2019 and 2020 results were similar; all traits were correlated. GWAS analysis demonstrated that six traits exhibited consistent and precise association signals. Of the latter, 104 were significant and detected in 43 genomic regions. By screening forage trait-associated single nucleotide polymorphisms and combining Manhattan map with genome annotation, signals were categorized according to functional annotations. One loci associated with fresh weight per plant (FWP) (chromosome 5; *Bnt05G007759*), two associated with FWPC (chromosome 13; *Bnt13G018582*, and *Bnt13G018583*), and two associated with leaf dry weight per plant (LDWP) and dry weight per plant (DWP) (chromosome 4; *Bnt04G005779* and *Bnt04G005780*), were identified. We describe forage trait candidate genes that are highly correlated with FWP and FWPC; *Bnt05G007759* may be involved in nitrogen metabolism, while *Bnt13G018582* and *Bnt13G018583* may encode TEOSINTE branch 1/CYCLOIDEA/proliferating cytokine 1 (TCP) domains. *Bnt04G005779* and *Bnt04G005780*, which may regulate growth and development, are highly related to LDWP and DWP. These genomic resources will provide a basis for breeding varieties.

## 1. Introduction

Ramie (China grass) is one of the oldest vegetable fiber crops in China, and its fibers have been used as textiles for over 4700 years in China [1]. However, as a fibrous crop application, only 5% of the whole plant is used, with the remaining 95% abandoned [2]. Consequently, many byproducts of ramie are not utilized well every year, except for fibers. Therefore, it is necessary to evaluate the feeding value of ramie to promote multifunctional development.

The demand for animal products has increased markedly and protein-rich feed shortages have consequently increased sharply [3]. To meet the ever-increasing requirement for feed, there is an urgent need to search for domestic plant resources with high nutrient content, such as ramie. Many studies have shown that fresh ramie leaves and shoot tips, which can be used as animal feed, have low fiber content and are rich in nutrients, such as crude protein (CP), lysine, and carotene [4,5,6,7]. Ramie is an unconventional forage resource with high biomass and rich in carbohydrates and minerals [8,9]. Toledo et al. reported that ramie could replace alfalfa and improve rabbit growth [6]. Tang et al. showed that there were no negative impacts on the growth of black sheep when ramie was used instead of alfalfa hay [10]. Therefore, it is necessary to study the forage characteristics of ramie and ramie varieties with high biological yields and protein content.

Individual plant height (IPH) and fresh weight per plant (FWP) are important factors influencing ramie biomass yield. CP and fiber components are two critical indices of forage quality that affect digestibility and nutritional intake during animal feeding [11]. In recent years, despite the use of ramie as feed, little progress has been made in research on feeding traits of ramie.

The aim of genome-wide association studies (GWAS) is to discern a large number of SNPs and identify trait-associated SNPs to reveal their biological mechanisms [12]. Compared to traditional genetic mapping methods, GWAS uses thousands of SNPs and linkage disequilibrium in the genome to identify quantitative trait loci (QTL) related to traits of interest. GWAS facilitates the analysis of complex trait loci in plants [13,14] and has been successfully used to detect plant morphological genetic loci [14], disease resistance [15,16], grain size [17], and many other agronomic traits [18]. SNPs significantly related to quantitative traits were obtained by GWAS and were associated with target candidate genes, which have been widely used in many plants. Twenty-two candidate genes were identified for resistance against *Verticillium* wilt in cotton using GWAS [19]. GWAS helped identify six candidate genes with pleiotropic effects on maize stem cell wall components [20]. Furthermore, a gene associated with arsenic accumulation in the leaves of *Arabidopsis* was determined by conducting GWAS [21]. GWAS has also been successfully applied to the detection of genetic loci associated with ramet number in ramie [22]. However, the molecular marker-selective breeding associated with the forage traits of ramie remains largely unclear, especially for GWAS analysis. Here, we present a comprehensive genomic variation map of ramie obtained by sequencing 301 landraces and cultivar accessions. Furthermore, to identify genetic markers associated with forage quality traits in ramie germplasm resources, which was a major objective of this study, we performed a GWAS with respect to six forage traits. Furthermore, candidate genes related to forage quality traits of ramie are also discussed. This study helps clarify the forage characteristics of ramie germplasms and provides candidate genes controlling the feeding quality traits of ramie.

## 2. Results

### 2.1. Phenotypic Trait Analysis

**Figure 1:**
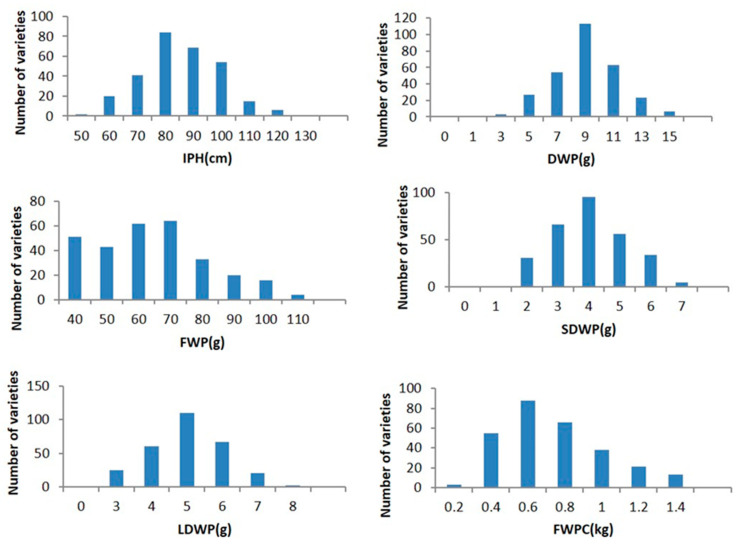
Frequency distribution of the 6 traits among the 301 ramie accessions (2019). Notes: IPH (individual plant height), FWP (fresh weight per plant), DWP (dry weight per plant), LDWP (leaf dry weight per plant), SDWP (stem dry weight per plant), and FWPC (fresh weight per clump).

**Figure 2:**
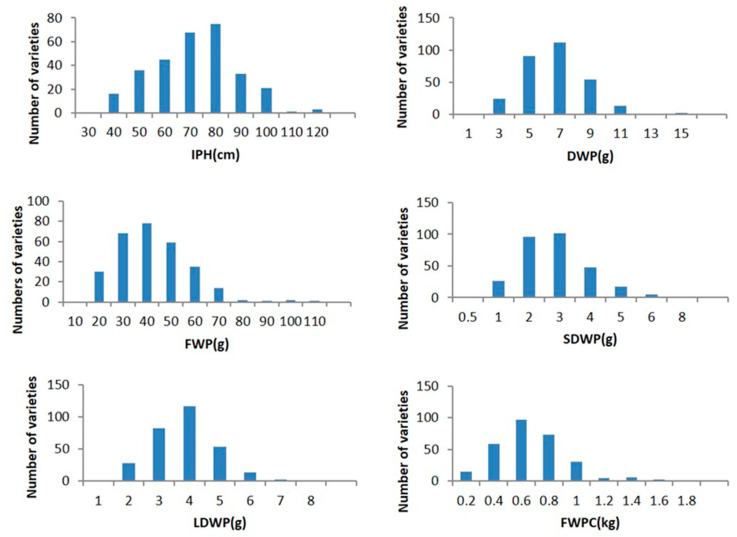
Frequency distribution of the 6 traits among the 301 ramie accessions (2020). Notes: IPH (individual plant height), FWP (fresh weight per plant), DWP (dry weight per plant), LDWP (leaf dry weight per plant), SDWP (stem dry weight per plant), and FWPC (fresh weight per clump).

A total of 301 ramie varieties were harvested on 10 June 2019, and 2020, and the traits related to forage were recorded for assessment. The results indicated that all traits showed tremendous phenotypic variation and continuous distribution in the population with the coefficient of variation ranging from 18.0% to 57.0% and were close to fitting a normal distribution (Figure 1 and Figure 2). Therefore, we considered these traits to be quantitative. In particular, the variation coefficient of fresh weight per clump (FWPC) was the highest in 2019 and that of IPH was the lowest in 2019. In addition, all trait variation coefficients were slightly lower in 2020 than they were in 2019 (Table 1). The coefficient of variation (CV) is the main index used to measure the degree of dispersion of population traits. A better estimate of the correlation between markers and traits can be obtained with a high degree of trait dispersion and large trait separation. In general, the coefficients of variation for these traits were relatively high and suitable for GWAS analysis.

Correlation analysis was performed to gain insight into the relationships between these traits (Table 2 and Table 3). The results for 2019 and 2020 are very similar, and all features are highly correlated. The correlations between traits were extremely significant (Table 2 and Table 3).

### 2.2. GWAS Analysis for Traits RELATED to Forage

The genetic structure of a population can markedly influence the power of a GWAS; thus, before performing the GWAS, we inferred the population structure using a subset of 1,433,616 SNPs without close linkages [23]. Our previous studies, through Bayesian cluster analysis and principal component analysis (PCA), show that our group has no obvious grouping, suitable for GWAS. We also analyzed the linkage disequilibrium (LD), and discovered that the average coverage density of SNP in this study was 45.45 bp, less than 0.65 kb of LD decay distance [24]. This indicated that the SNP density used in this study was sufficient to capture all genetic variations in the population.

**Figure 3:**
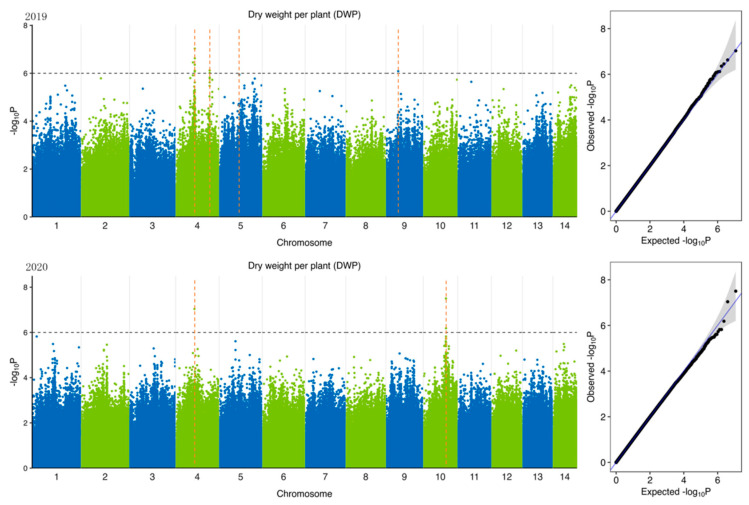
Manhattan plots of GWAS results for dry weight per plant (DWP) in 2019 and 2020. Notes: Blue and green dots represented SNP (single nucleotide polymorphism) sites on chromosomes, respectively. When the value of −log_10_ *p* was above 6 (gray line), the selected SNP site with significant aboriginality. Orange dotted lines represented the location of SNP loci.

**Figure 4:**
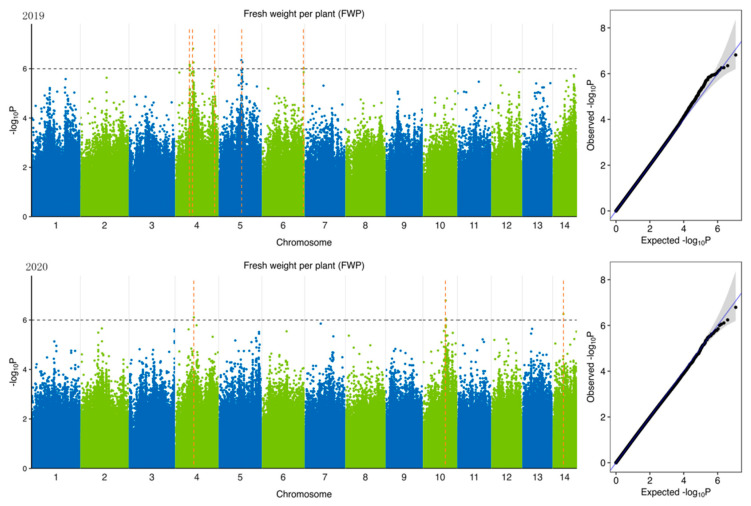
Manhattan plots of GWAS results for fresh weight per plant (FWP) in 2019 and 2020. Notes: Blue and green dots represented SNP (single nucleotide polymorphism) sites on chromosomes, respectively. When the value of −log_10_ *p* was above 6 (gray line), the selected SNP site with significant aboriginality. Orange dotted lines represented the location of SNP loci.

**Figure 5:**
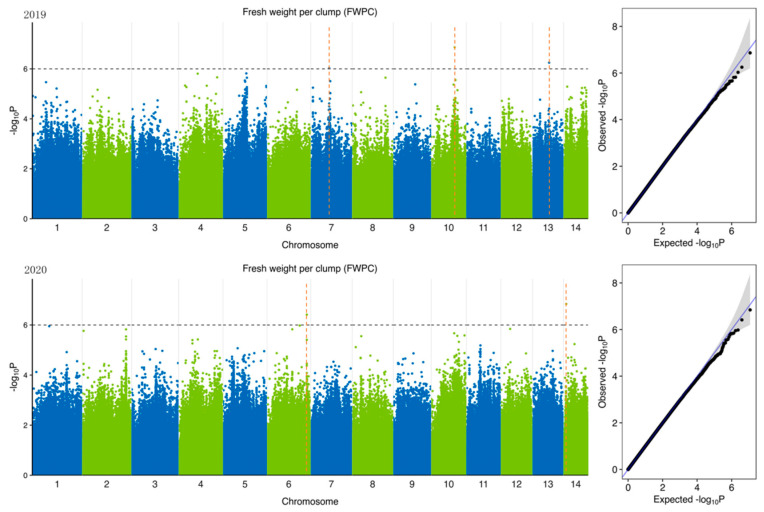
Manhattan plots of GWAS results for fresh weight per clump (FWPC) in 2019 and 2020. Notes: Blue and green dots represented SNP (single nucleotide polymorphism) sites on chromosomes, respectively. When the value of −log_10_ *p* was above 6 (gray line), the selected SNP site with significant aboriginality. Orange dotted lines represented the location of SNP loci.

**Figure 6:**
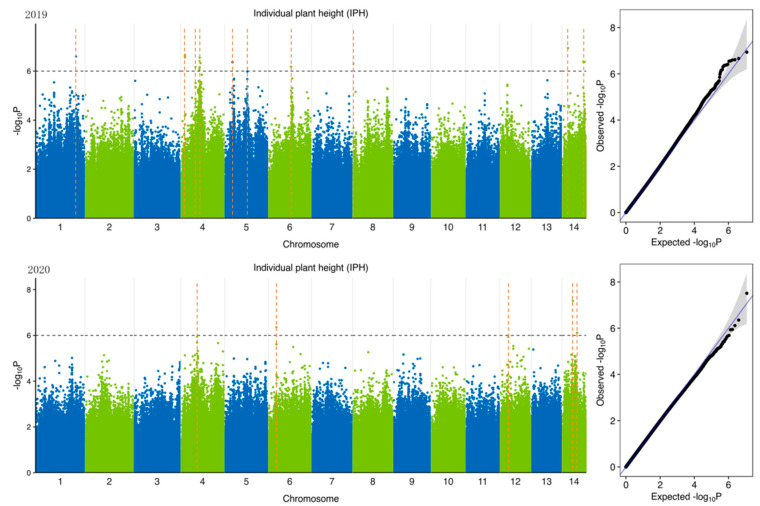
Manhattan plots of GWAS results for individual plant height (IPH) in 2019 and 2020. Notes: Blue and green dots represented SNP (single nucleotide polymorphism) sites on chromosomes, respectively. When the value of −log_10_ *p* was above 6 (gray line), the selected SNP site with significant aboriginality. Orange dotted lines represented the location of SNP loci.

**Figure 7:**
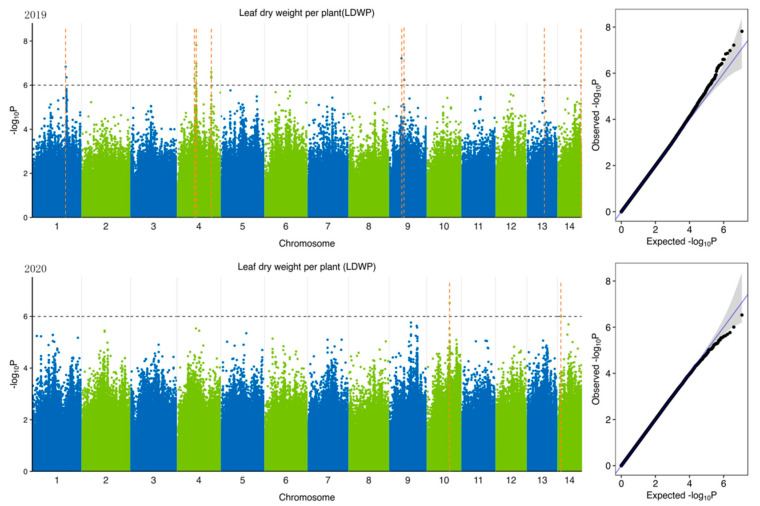
Manhattan plots of GWAS results for leaf dry weight per plant (LDWP) in 2019 and 2020. Notes: Blue and green dots represented SNP (single nucleotide polymorphism) sites on chromosomes, respectively. When the value of −log_10_ *p* was above 6 (gray line), the selected SNP site with significant aboriginality. Orange dotted lines represented the location of SNP loci.

**Figure 8:**
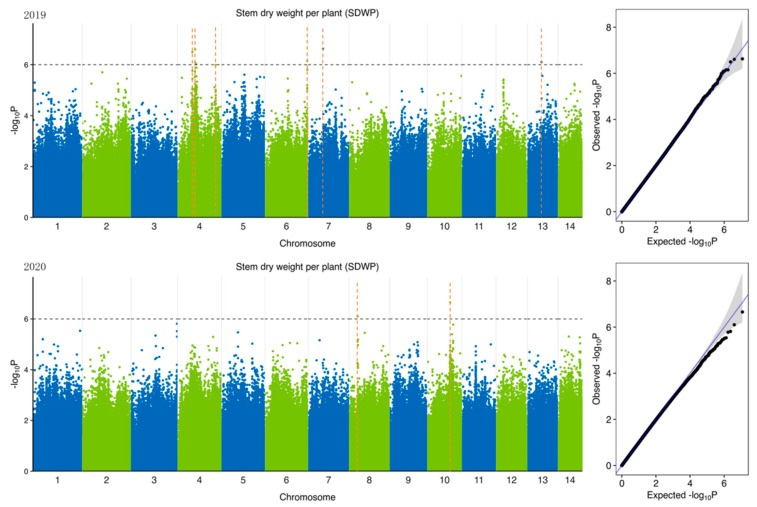
Manhattan plots of GWAS results for stem dry weight per plant (SDWP) in 2019 and 2020. Notes: Blue and green dots represented SNP (single nucleotide polymorphism) sites on chromosomes, respectively. When the value of −log_10_ *p* was above 6 (gray line), the selected SNP site with significant aboriginality. Orange dotted lines represented the location of SNP loci.

To identify potential candidate genes related to forage traits in natural populations of ramie accessions, according to 5,951,826 SNPs (Table 4) identified from 301 accession and 6 feeding traits of ramie, we performed GWAS of traits related to forage in ramie using Efficient Mixed-Model Association eXpedited (EMMAX). We determine the threshold according to the Q-Q plot (Figure 3, Figure 4, Figure 5, Figure 6, Figure 7 and Figure 8) on the right side of the figure. When −log_10_ *p*-value is 6, the statistical model conforms to the expected value. In total, 78 significant association signals were detected in 43 genomic regions (Figure 3, Figure 4, Figure 5, Figure 6, Figure 7 and Figure 8; Tables S2–S4). Of the loci identified, 18 were LDWP-associated, 17 were IPH-associated, 15 were FWP-associated, 12 were DWP-associated, 9 were SDWP-associated, and 7 were FWPC-associated. Notably, the SNP from chromosome 4 (position 9,261,790) was significantly associated with both the traits of LDWP and DWP, and it showed the largest signal associated with the LDWP (*p* = 1.5 × 10^−8^) among all association SNPs. In addition, there was another significant SNP on chromosome 4 (position 9,064,066), which was significantly correlated with the traits of LDWP and DWP, and was located between *Bnt04G005779* and *Bnt04G005780* genes.

### 2.3. Identification of Candidate Genes

When a −log_10_ *p*-value was > 6, SNPs above this threshold were deemed to be significantly associated with the target traits. We examined genes containing SNPs associated with forage traits and revealed that 78 forage trait-associated SNPs were located in 43 gene regions (including the genic region and upstream/downstream region within 20 kb) (Table S2–4). We categorized the 63 candidate genes according to functional annotations by screening SNPs closely associated with forage traits through the Manhattan map and combined these with genome annotation (Table S5). Next, we selected ten signals that may be related to forage traits. Our results revealed that among the genes containing SNPs associated with these six traits, one was associated with IPH, two with FWPC, three with FWP, four with DWP, three with LDWP, and two with SDWP (Table 5).

**Figure 9:**
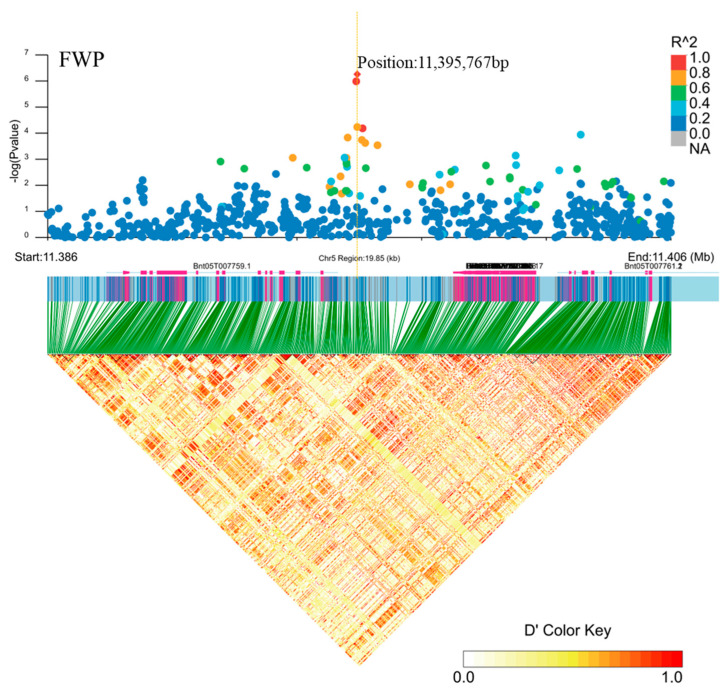
Candidate genes for FWP (fresh weight per plant) on chromosome 5. Manhattan plot (top) and linkage disequilibrium heatmap (bottom) of genomic region surrounding *Bnt05G007759*. A significant signal was associated with FWP on chromosome 5, as assessed using EMMAX (Efficient Mixed-Model Association eXpedited), and it was in *Bnt05G007759*. The graph shows genomic positions (*x* axis) and the respective significance expressed as −log_10_ *p* value (*y* axis). The genomic position spans ~20 kb on either side of the peak SNP (single nucleotide polymorphism), indicated by an orange dashed vertical line. The larger the R^2 (Coefficient of correlation) values (indicated in the legend on the top right), the stronger the degree of association. The red diamond indicates the associated SNP, and the lighter colors of the remaining markers reflect their successively lower R^2 values. D’ represents the correlation between different loci, the larger the value, the stronger the correlation and the higher the degree of linkage disequilibrium. The heat map showed that there was a strong linkage between the regions 11.386–11.406 Mb.

Annotated genes were searched within the interval associated with the SNP linkage disequilibrium block and within the physical position of each significant SNP. A peak strongly associated with FWP, identified on chromosome 5, was located in *Bnt05G007759* (position: 11,395,767 bp, Figure 9). Through genetic annotation, we found that *Bnt05G007759* has a critical domain, the MOSC N-terminal beta barrel (Table 5). Molybdenum cofactor sulfurase (MCSU) contains a key domain in the MOSC N-terminal β-barrel. Its main structural features are the β-barrel structure at the N-terminus of the MOSC domain and the strictly conserved cysteine residues at the extreme C-terminus [25]. MCSUs participate in processes such as nitrogen metabolism and the regulation of abscisic acid levels in plant tissues and are important enzymes for plant development and response to environmental queues [26]. Nitrogen is an essential nutrient for plant growth and development, and is a major factor limiting crop yield. Ramie is a crop with a very high nitrogen requirement. Biological yield is positively correlated with soil nitrogen levels, and FWP is an important factor in ramie biological yield. Therefore, there was a strong correlation between genes related to nitrogen metabolism and the FWP of ramie. Thus, we speculated that there is a correlation between MCSUs and ramie growth and development, and that *Bnt05G007759* might be a logical candidate for FWP.

**Figure 10:**
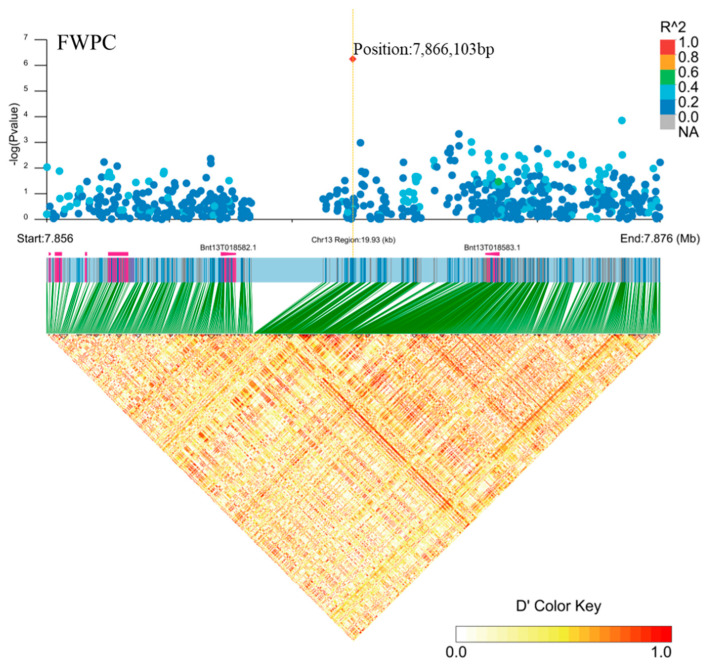
Candidate genes for FWPC (fresh weight per clump) on chromosome 13. Manhattan plot (top) and linkage disequilibrium heatmap (bottom) of genomic region surrounding *Bnt13G018582* and *Bnt13G018583*. A significant signal was associated with FWPC on chromosome 13, as assessed using EMMAX (Efficient Mixed-Model Association eXpedited), and it was between *Bnt13G018582* and *Bnt13G018583*. The graph shows genomic positions (*x* axis) and the respective significance expressed as −log_10_ *p* value (*y* axis). The genomic position spans ~20 kb on either side of the peak SNP (single nucleotide polymorphism), indicated by an orange dashed vertical line. The larger the R^2 (Coefficient of correlation) values (indicated in the legend on the top right), the stronger the degree of association. The red diamond indicates the associated SNP, and the lighter colors of the remaining markers reflect their successively lower R^2 values. D’ represents the correlation between different loci, the larger the value, the stronger the correlation and the higher the degree of linkage disequilibrium. The heat map showed that there was a strong linkage between the regions 7.856–7.876 Mb.

Among the signals associated with the FWPC trait, we found a particularly interesting association locus on chromosome 5 that was associated with the FWPC trait located between the genes *Bnt13G018582* and *Bnt13G018583* (position: 7866103bp, Figure 10). *Bnt13G018582* and *Bnt13G018583* belong to the TEOSINTE branch 1/CYCLOIDEA/proliferating cytokine 1 (TCP) transcription factor family, which participates in cell growth, leaf tissue proliferation, and branch growth [6,27,28]. The TCP family is a small plant-specific gene family that was first identified in 1999. The TCP domain is a specific transcription factor with a 59-amino acid basic helix-loop-helix motif [29]. FWPC is an important indicator of the biological yield of ramie. An increase in leaves and branches increases the biological yield of ramie. The TCP gene affects leaf and branching growth of plants; therefore, we speculate that *Bnt13G018582* and *Bnt13G018583* are potential candidate genes for the traits of FWPC.

**Figure 11:**
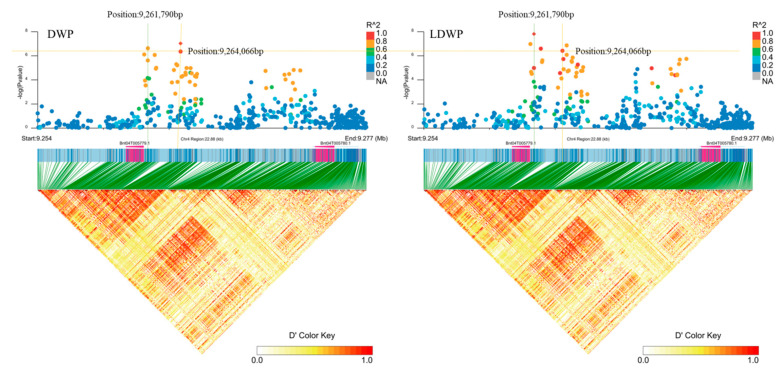
Candidate genes for DWP (dry weight per plant) and LDWP (leaf dry weight per plant) on chromosome 4. Manhattan plot (top) and linkage disequilibrium heatmap (bottom) of genomic region surrounding *Bnt05G007759*. A significant signal was associated with DWP (left) and LDWP (right) on chromosome 14, as assessed using EMMAX (Efficient Mixed-Model Association eXpedited), and it was between *Bnt04G005779* and *Bnt04G005780*. Besides, there is another significant signal associated with DWP (left) and LDWP (right) on chromosome 14, the signal is on *Bnt04G005779*. The graph shows genomic positions (*x* axis) and the respective significance expressed as −log_10_ *p* value (*y* axis). The genomic position spans ~23 kb on either side of the peak SNP (single nucleotide polymorphism), indicated by orange and green dashed vertical line. The larger the R^2 (Coefficient of correlation) values (indicated in the legend on the top right), the stronger the degree of association. The red diamond indicates the associated SNP, and the lighter colors of the remaining markers reflect their successively lower R^2 values. D’ represents the correlation between different loci, the larger the value, the stronger the correlation and the higher the degree of linkage disequilibrium. The heat map showed that there was a strong linkage between the regions 9.254–9.277 Mb.

In addition, we found two genes, *Bnt04G005779* and *Bnt04G005780* (position: 9,261,790 bp, 9,264,066 bp, Figure 11), which were significantly associated with both LDWP and DWP traits on chromosome 4. *Bnt04G005779* encodes a protein containing an F-box domain, whereas *Bnt04G005780* belongs to the F-box gene family. At the N-terminus of the F-box protein, there is an F-box domain of approximately 40–50 amino acids, that is mainly associated with the binding protein Skp1. The skeleton proteins Cullin1 and Rbx1 form the SCF complex, and at the C-terminus of the F-box protein, the SCF complex is usually the secondary structure that mediates the specificity of substrate recognition [30,31]. The majority of F-box gene-encoded proteins that play a role in the ubiquitin-protease pathway are SCF complexes. However, a small number of F-box proteins in the form of non-SCF complexes are also involved [32]. LDWP and DWP are important ramie biomass indicators. F-box genes are widely present in plants and are among the largest and fastest-evolving gene families. The biological functions of these gene families are diverse and are distributed among various plants. They play an important role in plant growth and development, biotic and abiotic stresses, and other processes. Therefore, we speculate that *Bnt04G005779* and *Bnt04G005780* are potential candidate genes for LDWP and DWP traits.

## 3. Discussion

### 3.1. Analyzing the Characteristics of Ramie and Related Indexes of Feeding Characters

Ramie, a perennial bast fiber crop widely grown throughout China, has been used as animal fodder because of its high fresh yield and leaves rich in nutrients including protein and eight amino acids essential for animal health [33]. It can be made into fresh fodder or dried into leaf meal; fresh leaves are used in the United States, Japan, Brazil, Spain, Colombia, and other countries as feed for cattle, chickens, and pigs [34]. The addition of ramie leaves to livestock and poultry diets has been shown to improve their production [5,35]. Toledo et al. found that using ramie as feed to replace 15% alfalfa enhanced the growth of rabbits [6]. There are an increasing number of reports on ramie feeding; however, to date, ramie feeding characteristic indices have not been standardized and there are very few research reports on the same. In this study, we investigated and analyzed six feeding traits of 301 ramie resources over a period of two years. Abundant phenotypic variation and heritability estimates are significant for successfully detecting the genetic basis of target traits. The CVs of six biological yield traits of forage ramie were all greater than 0.20, and three of them had CVs exceeding 0.40 (Table 1). The CVs observed in this study were generally higher than those reported in a previous study on ramie [22], suggesting that ramie has more abundant phenotypic variation in forage traits. This will provide a clear target for breeding forage ramie varieties.

### 3.2. Application of GWAS in Forage Ramie

It is important to study ramie genetic resources to breed new varieties and for global biodiversity conservation. GWAS of ramie traits is rare; candidate ramie genes were obtained using GWAS analysis for the first time [22]. However, our knowledge of the genetic variation and architecture of forage ramie traits is still limited. Forage characteristics are typical quantitative traits in ramie; CP, IPH, FWP, DWP, LDWP, SDWP, and FWPC are controlled by multiple genes. A previous study detected an association between SNPs and quantitative traits in forage ramie using an association population consisting of 301 core germplasms [22]. In this study, the core collections used for GWAS were obtained from different regions in China and represent the typical characteristics of various feed ramie varieties. In addition, all traits were continuously distributed in this population and were close to a normal distribution (Figure 1 and Figure 2), with high levels of genotypic and phenotypic diversity (Table 1) suitable for GWAS analysis.

### 3.3. Putative Candidate Genes Associated with Forage Quality

GWAS analysis was used to determine the number of target regions that putatively controlled important agronomic traits. Among the nine annotated genes associated with forage quality traits, three—*Bnt13G018582, Bnt13G018583*, and *Bnt05G007759*—were identified by LD block and GWAS analyses (Table 5). The genes *Bnt13G018582 and Bnt13G018583* contain a TCP domain. It has been postulated that TCP transcription factors regulate plant growth and development by mediating environmental factors and hormone signaling pathways [36,37,38]. TCPs have been studied extensively with regard to the regulation and control of leaf morphology by promoting the differentiation of leaf pavement cells via both auxin- and auxin-independent pathways. TCP4 restricts the cell number and final size of leaves in *Arabidopsis* [39]. He et al. demonstrated that TCP2 regulates leaf morphogenesis via a mechanism involving cell proliferation in *Arabidopsis* [40]. Previous studies have shown that DELLAs directly regulate the activity of the plant-specific class I TCP transcription factor family, which are the key regulators of cell proliferation [41]. Daviere et al. demonstrated that class I TCP factors directly bind to the promoters of core cell-cycle genes in *Arabidopsis* inflorescence shoot apices, while DELLAs block TCP function by binding to their DNA-recognition domain [38]. In our study, we used GWAS analysis to obtain two candidate genes, *Bnt13G018582* and *Bnt13G018583*, which are associated with critical forage traits, FWPC of ramie, and contain a TCP domain. Therefore, we suggest that these genes may be involved in leaf growth and plant height of forage ramie; however, their specific function needs to be tested further and verified.

MCSUs participate in processes such as nitrogen metabolism and the regulation of abscisic acid levels in plant tissues and are important enzymes for plant development and response to environmental cues. There are three critical domains in all MCSUs: aminotransferase class V, the MOSC N-terminal beta barrel, and MOSC [26]. In our study, we identified the candidate gene *Bnt05G007759* using GWAS analysis. This gene contains an MOSC N-terminal beta barrel domain, may be involved in nitrogen metabolism in ramie, and is related to forage traits of ramie.

Plant F-box proteins regulate many key plant physiological processes, including hormone regulation, light signal transduction, self-incompatibility, floral organ development, pathogen invasion resistance, and specific recognition of degraded proteins [38]. The first F-box protein discovered was UFO, and UFO genes play an essential role in flower meristem and organ development [42]. The *Arabidopsis* F-box gene At PP2-B11 plays an important role in drought stress as a negative regulator, reducing plant tolerance to drought [43]. The At2g02360 (F-box-Nicta) gene in *Arabidopsis thaliana* encodes a sugar-condensing F-box protein. Overexpression of F-box-Nicta in transgenic *A. thaliana* can reduce the bacterial colonization rate after Pst DC3000 infection and reduce leaf damage [44], and FBX92 affects plant leaf size [45]. Therefore, we believe that the *Bnt04G005779* and *Bnt04G005780* genes may ensure the normal operation of life activities through multiple coordinated nodal mechanisms; however, their specific functions require further testing and verification.

The identification of target genes can be used in future functional and molecular breeding studies. These new genomic resources will promote research on genome evolution, perennation mechanisms, and other traits of forage ramie. This, in turn, will facilitate the breeding of improved forage to benefit human society.

## 4. Materials and Methods

### 4.1. Plant Materials

In the present study, 301 core germplasms of ramie used for SNP development were cultivated at the Institute of Bast Fiber Crops, Chinese Academy of Agricultural Sciences. The germplasms were collected from China, India, and Cuba (Table S1). All accessions were used for asexual propagation by tender stem cutting in the testing field of Yuanjiang, Hunan Province, China (112°33′ N, 28°16′ E), in 2016. We conducted field planting trials according to a randomized block design. There are 2 rows (2 repetitions) for each variety, 6 plants in each row, the row length is 3.6 m, and the row spacing is 1 m. We selected core germplasms with abundant phenotypic variation that could represent ramie germplasm resources as suitable accessions for GWAS.

In May 2019 and 2020, fresh leaves of 301 ramie accessions were collected twice, placed in liquid nitrogen in an icebox, and frozen at −80 °C until genomic DNA was extracted for sequencing.

### 4.2. Phenotype Evaluation

The traits related to forage, such as IPH (straight length from ground to main stem tip), FWP (fresh weight of each plant cut off from the ground), DWP (dry weight measured per plant), LDWP (dry weight of leaves per plant after drying), stem dry weight per plant (SDWP: dry weight of stem after drying), and FWPC (total fresh weight of several individual plants of one ramie clump), were recorded for assessment. According to the normal cultivation method, 301 accessions were harvested on 10 June 2019, and 10 June 2020. Afterwards, the traits were investigated in the second growing season of each year, when the ramie grew to about a meter high.

### 4.3. Statistical Analyses

Multiple comparisons of all parameters were conducted using one-way analysis of variance (ANOVA) and Tukey’s honest significant difference test.

Descriptive statistics were computed using SPSS 22.0. Microsoft Excel 2010 was used to determine the frequency distribution for each trait.

### 4.4. Population Structure and LD Analysis

All SNPs from ramie accessions were filtered using the PLINK software (version: 1.9; parameters -indep-pairwise 50 5 0.5) [46], generating a subset of SNPs without close linkages. Investigation of population structures was carried out using ADMIXTURE software (version: 1.3.0) [47], and it was run 10 times for each K value with varying random seeds; the Q-matrices were aligned using pong software and were clustered based on similarity [48]. Thereafter, the matrices from the largest cluster were averaged to produce a final matrix of admixture proportions. A PCA was performed using the smartPCA program of EIGENSOFT software (version: 6.1.4) [49]. To measure LD levels in groups of landraces and the bred cultivars, r^2^ of each allele was estimated using PopLDdecay (version: 3.34) with the following parameters: −MAF 0.05 −Miss 0.2 −MaxDist 300 [50]. Average r^2^ values were calculated for each distance.

### 4.5. GWAS Analysis

Genome sequence was performed for 301 accessions using the next-generation Illumina HiSeq platform. Sequencing and analysis were conducted by OE Biotech Co., Ltd. (Shanghai, China). Raw sequence reads were filtered to produce clean reads that were aligned to the ramie genome (downloaded from NCBI; accession no. PRJNA663427) using BWA (version 0.7.15), allowing no more than 4% mismatch and one gap [51]. The alignment results were converted to bam format using SAMtools (version: 1.9) [52], and duplicated reads were removed using the picard package. GATK (version 3.8.1) [53] was used to identify SNPs and indels. GWAS analysis was performed based on 5,951,826 SNP data of the population (accession number GVM000103 in the Big Data center at the BIG). All SNPs from ramie accessions were filtered using the PLINK software (version: 1.9; parameters-indep-pairwise 50 5 0.5; Purcell et al., 2007), generating a subset of SNPs without close linkages for population structure analysis [46]. Efficient Mixed-Model Association eXpedited (EMMAX) software was used to perform GWAS using SNP genotypes and phenotype matrices under principal component analysis and kinship as covariations [54,55], and GEC software was used to calculate the effective number of independent markers and significant *p*-value threshold [56]. Manhattan and quantile-quantile plots were created using the R package CMplot (https://github.com/YinLiLin/RCMplot (accessed on 8 December 2020).). According to genome annotation, SNPs were divided into five regions: exon, intron, upstream, downstream, and intergenic. After screening for counterfeit SNPs, high-quality and significantly correlated SNPs were obtained, and the threshold was log_10_ *p*-value ≥ 6.

### 4.6. Prediction of Candidate Genes

BLAST analysis was conducted on the ramie genome using favorable SNP sequences [57], and determined the SNPs related to forage traits. The size of the site interval was estimated based on LD block analysis, and the genes in the region near the site were obtained, and the functional annotation of these genes was performed. According to the functional annotation, we can roughly determine which genes may be related to the traits we are concerned with and enter the subsequent analysis.

## Figures and Tables

**Table 1:** Analysis of variation coefficient of forage quality traits in the panel of 301 ramie accessions.

Traits	Years	Mean	STDEV	Skewness	Kurtosis	CV
IPH	2019	80.41041	14.45221	0.08226	−0.09009	0.1797
	2020	66.93907	16.22265	0.0334	−0.27588	0.2426
FWP	2019	58.87703	18.55376	0.167087	−0.44334	0.3151
	2020	36.75665	15.74601	0.577069	1.410746	0.4284
DWP	2019	8.035631	2.276232	0.011917	−0.10541	0.2833
	2020	5.683078	2.034604	0.670854	0.957744	0.3580
LDWP	2019	4.494491	1.159356	−0.70925	1.836823	0.2580
	2020	3.321628	1.009748	0.453133	0.563682	0.3040
SDWP	2019	3.510219	1.171264	0.238948	−0.33708	0.3337
	2020	2.36334	1.121519	1.085063	2.395851	0.4745
FWPC	2019	0.69955	0.398916	2.058627	6.861975	0.5702
	2020	0.577711	0.263481	1.026716	2.199923	0.4561

Notes: IPH (individual plant height); FWP (fresh weight per plant); DWP (dry weight per plant); LDWP (leaf dry weight per plant); SDWP (stem dry weight per plant); FWPC (fresh weight per clump). Mean: mean value; STDEV: standard deviation; CV: coefficient of variation.

**Table 2:** Correlation coefficients among the 6 traits in the 302 ramie accessions (2019).

2019	IPH	FWP	DWP	LDWP	SDWP	FWPC
IPH						
FWP	0.794 **					
DWP	0.796 **	0.840 *				
LDWP	0.633 **	0.748 **	0.933 **			
SDWP	0.877 **	0.891 **	0.926 **	0.757 **		
FWPC	0.701 **	0.589 **	0.557 **	0.416 **	0.645 **	

* and ** indicate significant differences at *p* = 0.05 and *p* = 0.01, respectively. Notes: IPH (individual plant height), FWP (fresh weight per plant), DWP (dry weight per plant), LDWP (leaf dry weight per plant), SDWP (stem dry weight per plant), and FWPC (fresh weight per clump).

**Table 3:** Correlation coefficients among the 6 traits in the 302 ramie accessions (2020).

2020	IPH	FWP	DWP	LDWP	SDWP	FWPC
IPH						
FWP	0.825 **					
DWP	0.850 **	0.915 **				
LDWP	0.720 **	0.837 **	0.949 **			
SDWP	0.894 **	0.908 **	0.959 **	0.820 **		
FWPC	0.704 **	0.826 **	0.795 **	0.711 **	0.803 **	

** indicates significant differences at *p* = 0.05 and *p* = 0.01, respectively. Notes: IPH (individual plant height), FWP (fresh weight per plant), DWP (dry weight per plant), LDWP (leaf dry weight per plant), SDWP (stem dry weight per plant), and FWPC (fresh weight per clump).

**Table 4:** Distribution of SNPs in ramie genome.

Chr.	SNPs	Intergenic	CDS	Intron	NS SNPs	S SNPs
Chr1	532,773	219,654	44,626	97,484	20,658	23,968
Chr2	481,607	191,354	43,443	93,941	20,173	23,270
Chr3	462,186	186,239	42,467	96,791	20,737	21,730
Chr4	493,655	203,277	43,274	94,759	20,664	22,610
Chr5	456,788	185,700	43,097	87,148	20,447	22,650
Chr6	447,197	150,528	44,799	88,657	20,438	24,361
Chr7	454,335	227,789	33,706	68,160	15,682	18,024
Chr8	391,132	178,009	31,695	65,775	14,691	17,004
Chr9	440,598	209,133	32,741	78,206	15,541	17,200
Chr10	348,653	130,303	36,950	68,202	17,607	19,343
Chr11	418,597	196,178	32,787	77,175	15,233	17,554
Chr12	337,679	141,539	29,983	72,029	14,708	15,275
Chr13	359,036	152,503	35,334	73,229	16,874	18,460
Chr14	288,534	133,000	23,759	48,307	10,490	13,269
Scaffold	39,056	10,782	4196	6718	2008	2188
Total	5,951,826	2,515,988	522,857	1,116,581	245,951	276,906

Notes: Chr.: chromosome number; SNPs: single nucleotide polymorphisms; CDS: conserved domains; NS SNPs: non-synonymous SNPs; S SNPs: synonymous SNPs.

**Table 5:** Association signal-located genes with gene annotation in forage traits.

Chromosome Number	Gene	Associated Signal			Signal Position in Gene	Annotation
		Trait	SNP Position	−log _10_ *p* Values		
Chr13	*Bnt13G018582*	FWPC	7866103	6.3	Intergenic region	TCP domain-containing protein
Chr13	*Bnt13G018583*	FWPC	7866103	6.3	Intergenic region	TCP domain-containing protein
Chr14	*Bnt14G019331*	LDWP	1803576	6.0	Upstream gene variant	Transposase-associated domain
Chr04	*Bnt04G005762*	DWP	9087683	7.0	Intergenic region	Cation transport
		FWP	9087683	6.1	Intergenic region	
		IPH	9100978	6.5	Downstream gene variant	
Chr04	*Bnt04G005636*	FWP	7066661	6.2	Intron variant	CSC1-like protein
		SDWP	7110697	6.5	Intergenic region	
Chr04	*Bnt04G005637*	FWP	7110697	6.1	Intergenic region	Wall-associated receptor kinase-like 9
		SDWP	7110697	6.5	Intergenic region	
Chr05	*Bnt05G007759*	FWP	11395767	6.3	Upstream gene variant	MOSC, N-terminal beta barrel
Chr04	*Bnt04G005779*	LDWP	9261790	7.8	Downstream gene variant	F-box domain-containing protein
		LDWP	9264066	6.9	Intergenic region	
		DWP	9264066	7.0	Intergenic region	
		DWP	9261790	6.6	Downstream gene variant	
Chr04	*Bnt04G005780*	LDWP	9264066	6.9	Intergenic region	F-box protein
		DWP	9264066	7.0	Intergenic region	

Notes: We examined genes containing SNPs (single nucleotide polymorphisms) associated with traits and selected 10 signals that might be related to forage traits. Among the SNPs-containing genes associated with FWPC, FWP, LDWP, and DWP traits, five genes showed significant expression differences in functional annotation, and were selected and marked in red. MCSUs participate in processes such as nitrogen metabolism and the regulation of abscisic acid levels in plant tissues and are important enzymes for plant development and response to environmental queues. TCP transcription factor family, which participates in cell growth, leaf tissue proliferation, and branch growth. F-box genes are widely present in plants and are among the largest and fastest-evolving gene families. They play an important role in plant growth and development, biotic and abiotic stresses, and other processes.

## Data Availability

The variation data reported in this paper have been deposited in the Genome Variation Map (GVM) of the Big Data Center, Beijing Institute of Genomics (BIG), Chinese Academy of Science, under accession number GVM000103 at https://bigd.big.ac.cn/gvm/getProjectDetail?project=GVM000103 (accessed on 8 December 2020).

## Supplementary Materials

The following supporting information can be downloaded at: https://www.mdpi.com/article/10.3390/plants11111443/s1, Table S1: Basic information for 301 ramie accessions; Table S2: SNPs associated with six traits in ramie population, the yellow marker indicates the selected candidate gene and the red marker indicates the possible candidate gene; Table S3: SNPs associated with six traits in ramie population; Table S4: Genomic regions associated with forage trait in ramie. Table S5: DNA repair RAD52-like protein 1, mitochondrial OS = Arabidopsis thaliana OX = 3702 GN = RAD52-1 PE = 1 SV = 1.
